# Molecular mechanisms of drought resistance using genome-wide association mapping in maize (*Zea mays* L.)

**DOI:** 10.1186/s12870-023-04489-0

**Published:** 2023-10-06

**Authors:** Zhang Ningning, Liu Binbin, Ye Fan, Chang Jianzhong, Zhou Yuqian, Wang Yejian, Zhang Wenjie, Zhang Xinghua, Xu Shutu, Xue Jiquan

**Affiliations:** 1 Key Laboratory of Biology and Genetic Improvement of Maize in Arid Area of Northwest Region, College of Agronomy, Northwest A&F University, Yangling, Shaanxi, 712100 China; 2 Agricultural University of Shanxi, Taiyuan, Shanxi, 030600 China; 3 Crop Institute of Gansu Academy of Agricultural Sciences, Lanzhou, Gansu, 730000 China; 4 Institute of Grain Crops, Academy of Agricultural Sciences of Xinjiang, Urumqi, Xinjiang, 830000 China; 5 Crop Institute of Ningxia Academy of Agricultural Sciences, Yinchuan, Ningxia, 750000 China

**Keywords:** Maize, Genome-wide association studies, Drought resistance index, Candidate genes

## Abstract

**Background:**

Drought is a critical abiotic stress that influences maize yield and reduces grain yield when it occurs at the flowering or filling stage. To dissect the genetic architecture of grain yield under drought stress (DS), a genome-wide association analysis was conducted in a maize population composed of diverse inbred lines from five locations under well-watered and DS conditions at flowering in 2019 and 2020.

**Results:**

Using a fixed and random model circulating probability unification model, a total of 147 loci associated with grain yield or the drought resistance index (DRI) were identified, of which 54 loci were associated with a DRI with an average phenotypic variation explanation of 4.03%. Further, 10 of these loci explained more than 10% of the phenotypic variation. By integrating two public transcriptome datasets, 22 differentially expressed genes were considered as candidate genes, including the cloned gene *ZmNAC49*, which responds to drought by regulating stomatal density. Enrichment and protein interaction network showed that signaling pathways responded to drought resistance, including jasmonic acid and salicylic acid, mitogen-activated protein kinase, and abscisic acid-activated. Additionally, several transcription factors involved in DS were identified, including basic leucine zipper (*GRMZM2G370026*), NAC (*GRMZM2G347043*), and ethylene-responsive element binding protein (*GRMZM2G169654*).

**Conclusions:**

In this study, we nominated several genes as candidate genes for drought resistance by intergrating association maping and transcription analysis. These results provide valuable information for understanding the genetic basis of drought tolerance at the mature stage and for designing drought-tolerant maize breeding.

**Supplementary Information:**

The online version contains supplementary material available at 10.1186/s12870-023-04489-0.

## Introduction

Water scarcity and unpredictable droughts are posing a threat to corn production worldwide [1, 2]. As the Earth’s population increases, water pollution and climate change further aggravate the already declining water availability, which has caused many social problems and economic losses [3–5]. The water resource development report indicates that global water consumption has now increased threefold compared to that of the past 50 years, and the rising trend in agricultural water demand over the past few decades further confirms this situation [6, 7]. According to the data from the 2013 Intergovernmental Panel on Climate Change, the average surface temperature of the earth has increased by 0.85 ℃ over the past 130 years, resulting in frequent occurrences of extreme weather phenomena, such as droughts, floods, heat waves, strong winds, and thunderstorms, and drought is considered as the maximum limiting factor for crop yield [8, 9].

According to statistics from the United Nations Food and Agriculture Organization ( www.FAO.org ), maize is not only the major crop in China since 2011, but also the major cereal crop in the world. In addition, it is widely used in animal husbandry and is known as the “king of feed” [10–15]. The Corn grain can serve as an important raw material and industrial base for the development of new energy sources worldwide, and its utilization has significant practical value [16]. Drought at different growth stages in maize will decrease maize production by affecting the growth and development through different regulatory mechanisms. Drought causes yield reductions ranging from 9.3% to 35.1% in China [17] and the 2012 drought in the U.S. led to decreased grain yield (GY) by 21% compared to that of the previous 5 years, with an average country-yield of 7.7 mg ha^-1^ [18]. The drought tolerance level of maize is mainly assessed by the performance of dry matter production under water stress [19]. Therefore, selecting maize germplasm resources with high drought resistance is an important strategy to improve yield.

Plant drought tolerance is a complex trait regulated by numerous quantitative trait loci (QTL), with relatively small effects [9]. A good variety with high drought tolerance contains an assembly of favorite alleles. Therefore, identification of functional genes or markers closely associated with the regulation of drought tolerance is a crucial step in genomics-assisted plant breeding. To reveal the genetic architecture of yield and secondary traits in maize, most linkage mapping studies have reported QTL associated with drought tolerance [20]. Many single nucleotide polymorphisms (SNPs) associated with drought resistance have been identified in genome-wide association studies (GWASs) of multiple associated populations. The association panel consists of 350 tropical and subtropical inbred maize lines, which revealed 33 candidate genes associated with GY and related secondary traits under well-watered (WW) and drought stress (DS) conditions [19]. In addition, a GWAS using 240 inbred lines detected 52 candidate genes associated with seven agronomic traits, including yield (GY) and related secondary traits, under both WW and DS conditions [21]. These genetic analyses provide abundant information on functional gene clones. There is a significant correlation between variation in the *ZmVPP1* gene and drought tolerance in maize seedlings, and transgenic maize plants expressing *ZmVPP1* exhibit enhanced drought tolerance [22]. Recently, a small inverted-repeat transposable element of 82 base pairs in length inserted in the *ZmNAC111* promoter was shown to be associated with drought tolerance in maize seedlings [23]. *ZmMPKL1* regulates homeostasis of abscisic acid (ABA) and mediates the response of maize seedlings to DS by encoding a functional kinase [24]. However, most genes cloned in previous studies were analyzed and cloned based on survival rates at the seedling stage, whereas few genes related to drought resistance were cloned at the maturity stage. A recent association study in maize using a natural-variation population demonstrated a significant correlation between the expression level of *ZmEXPA4* and the increase in the anthesis and silking intervals (ASI) caused by drought. These results suggest that regulating the expression of *ZmEXPA4* using drought-induced promoters can potentially reduce the negative effect of drought on ASI [25].

In addition, with the development of high-throughput phenotyping, metabolomics, proteomics, and transcriptomics, many relevant parameters have been utilized to assess drought response; these parameters are valuable resources for genetic analyses aimed at identifying genes involved in drought regulation [10–15]. However, most of these studies focused on the seedling stage and were rarely related to drought tolerance of mature plants. Therefore, it is necessary to conduct genetic analyses of DS during the mature stage in the field. Here, a GWAS of drought tolerance was conducted using GY and related indices under different water treatment (WW and DS) at flowering time. The aim of the study was to identify associated SNPs and candidate genes to understand the genetic basis of drought resistance for future research.

## Materials and methods

### Plant materials and growth conditions

For the 201 maize inbred lines adopted in this study, except for six public inbred lines (PH6WC, PH4CV, Zheng58, Chang7-2) that were regarded as controls, the rest were selected and bred by the institutions involved in this study (Table S1). Due to the collected materials were newly bred by breeders, some will be eliminated or added by comprehansive envaluated. The number of inbred lines were used in 2019 was 115 and 180 in 2020, with 94 common inbred lines termed AM201, AM115, and AM180, respectively. AM115 and AM180 were planted with two replications under WW and DS in five locations in China: including Yulin (YL; 109°45'N, 38°16'E) in Shaanxi, Yinchuan (YC; 10°06'N, 37°43'E) in Ningxia, ZhangYe (ZY; 100°48'N, 38°93'E) in GanSu, Urumqi (UR; 87°61'N, 43.79'E) in Xinjiang, and Taiyuan (TY; 87°61'N, 43°79'E) in Shanxi. There was a 2 m-wide isolation belt between the WW and DS treatments. At each location, all inbred lines for each treatment were planted in two-row plots using a split block design, with a 5 m row length and 0.6 m row intervals, and at plant density of 75,000 plants ha^-1^. Normal irrigation ensured sufficient water supply during the entire growth period. DS was performed using field water at vegetative stages V13 to VT; during these stages, irrigation was applied when the relative water content of soil reached 50–60%, while normal irrigation was provided during the other growth periods. All other field management practices followed local practices.

### Phenotype data collection and analyses

GY (kg hm^-2^) was adjusted to a 14% moisture content. The number of effective plants in each plot was determined before harvest and the ears of all inbred lines in each plot were harvested. A PM-8188 grain moisture tester (Kett Electric Laboratory, Japan) was used to determine the kernel water content at harvest and to convert the yield per unit area. The formula is as follows:

$$GY=\frac{{W}_{dry}\times N\times 666.67\times (1-M)}{A\times (1-14\%)}$$

Where GY is the grain yield (kg hm^-2^); *W*_*dry*_ is the dry grain weight per ear (kg); N is the number of effective plants; *A* is the area of the two-row plot (m^2^); and *M* is the actual measurement of grain moisture content. Using the R package lme4 [26, 27], the best linear unbiased prediction (BLUP) values were calculated for AM115, AM180, and AM201 and termed 2019DS, 2019WW, 2020DS, 2020WW, 201DS, and 201WS, respectively. The drought resistance index (DRI) was calculated according to the related BLUP values from each association panel and termed 2019DRI, 2020DRI, and 201DRI, respectively. The detailed formula for the DRI is as follows, according to [28]:

$$\begin{array}{c}\mathrm{DC}=\mathrm{GYS}.\mathrm{T}/\mathrm{GYS}.\mathrm{W}\\ DRI=C\times (GYS.T/GYM.T)\end{array}$$

Where *DC* is the drought coefficient; *GYS.T* is the yield of the tested inbred lines under DS treatment, *GYS.W* is the yield of the tested inbred lines under WW treatment, and $GYM.T$ is the average yield of all tested materials under DS. Basic descriptive analysis was performed using the SPSS v.22 software (IBM corp., Armonk, NY, USA). Phenotypic variation in yield traits was evaluated using analysis of variance. Genotype (G), environment (E), the interaction between genotype and environment (G $\times$ E), and replication were fitted with a general linear model as follows:

$${y}_{ijk}=\mu +{G}_{i}+{E}_{j}+{G}_{i}\times {E}_{j}+R{\left(E\right)}_{jk}+{\varepsilon }_{ijk}$$

Where ${y}_{ijk}$ is the phenotypic value of the *i*th inbred line from the *k*th replication in the *j*th environment; $\mu$ is the overall mean of a trait; ${G}_{i}$ is the genetic effect of the *i*th inbred line; ${E}_{j}$ is the environmental effect of the *j*th environment; ${G}_{i}\times {E}_{j}$ is the interaction between genotype and environment for the *i*th inbred line and the *j*th environment; $R{\left(E\right)}_{jk}$ is the *k*th replication within the *j*th environment; and ${\varepsilon }_{ijk}$ is the residual error. The formula for calculating broad-sense heritability (H^2^) is as follows:

$${H}^{2}={ \sigma }_{\mathrm{g}}^{2}/\left({ \sigma }_{\mathrm{g}}^{2}+\frac{{\sigma }_{ge}^{2}}{n}+\frac{{\sigma }_{e}^{2}}{nr}\right)$$

Where ${\sigma }_{\mathrm{g}}^{2}$, ${\sigma }_{\mathrm{ge}}^{2}$, and ${\sigma }_{e}^{2}$ represent the genotypic variance, the G × E variance, and the residual error variance, respectively, and n and r are respectively the mean number of environments and replications that combine location and years as a random effect [29, 30].

### DNA extraction and genotyping

At the five-leaf stage, 10 young leaves were mixed-sampled and frozen at -80 ℃ for DNA extraction using a modified cetyltrimethyl ammonium bromide (CTAB) method [31, 32]. All DNA samples were submitted to the Beidahuang Company (Beidahuang Kenfeng Seed Industry Co., Ltd., Harbin, China) for genotype detection using the Maize 6H60K chip, which was independently developed by the Maize Research Center of the Beijing Academy of Agriculture and Forestry Sciences. A total of 42,974 markers were considered sufficiently robust and consistent for use in this analysis and were screened for missing values > 0.25 and minor allele frequency (MAF) < 0.05. This led to further analysis using 42,003 SNPs.

### Population structure and linkage disequilibrium (LD) analyses

The population structure was inferred using the PuTTY Link (PLINK) version 1.90 [33] and ADMIXTURE version 1.3.0 software [34]. The subgroup parameter value K was set to 1–20, and the best cross-validation error was determined by running the ADMIXTURE software K value, which was best when the cross-validation error rate was closest to zero. The Q-matrix was used as a covariance matrix for the association analysis. For AM201, an unweighted pair group method with arithmetic mean tree with 1000 bootstraps was constructed using the MEGA software (v7.0) [35] with a set of 42,003 high-quality SNPs. Principal component analysis (PCA) and kinship matrix analysis were performed using the LDAK5 [36] tool on the high-quality SNPs. The output of LDAK5 was fed as input into the RStudio software to graphically display the PCA and kinship matrix results, and the LD decay distance was calculated using the PopLDdecay software [37].

### Genome-wide association study

GWAS was conducted for GY and DRI using a fixed and random model of circulating probability unification (FarmCPU) in the R software [38]. Manhattan plots were generated using the CMplot package in R. Due to the stringent threshold calculated by the Bonferroni correction, the GWAS threshold was set as -log(*p*) = 3.5 to declare significant associations, which were determined based on the Q-Q plots and distribution of *p* values. Candidate genes were predicted according to the LD decay distance, which was 150 kb (*r*^2^= 0.2) for AM201. Here, the MaizeGDB database based on the B73 RefGen_v3 genome (https://maizegdb.org/gbrowse/maize_v3) was used as a reference, because the SNP chip data were designed and the transcripptomic data used later were calculated using this verb.

### Functional annotation of genes

Two public transcriptome datasets (Gene Expression Omnibus [GEO] accession numbers: GSE132113 and GSE71723), including three tissues (leaf, ear, and kernel) at four developmental stages (V12, V14, V18, and R1) and three tissues (leaf, ear, and tassel branch) at two stages (V9 and 5DAP) under DS were integrated using the National Center for Biotechnology Information (NCBI). Genes were regarded as differentially expressed using the cutoff criterion of log2Fold change ≥2. Candidate genes were clustered and visualized using the R-package pheatmap (http://www.r-project.org/). To annotate the potential functions of candidate genes, Gene Ontology (GO) analysis was performed using the Database for Annotation, Visualization and Integrated Discovery website (https://david.ncifcrf.gov/tools.jsp). Kyoto Encyclopedia of Genes and Genomes (KEGG) pathway [39–41] analysis was performed using the cluster Profile package in R [42]. The search tool for the retrieval of interacting genes/proteins (STRING, v11.0; https://string-preview.org/) was used to analyze the protein interaction network, whereas the Cytoscape software (V3.8.0; http://www.cytoscape.org/download.php) was used to visualize the protein interaction network [43].

## Results

### Evaluation of phenotypic variation

**Fig. 1:**
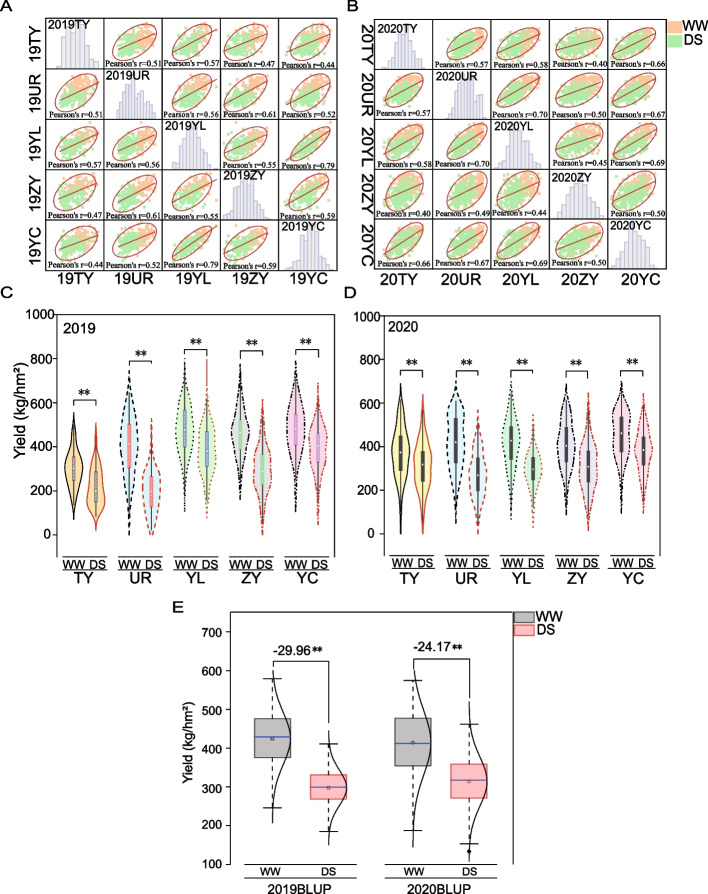
Phenotypic performance of yield traits in different populations across multiple environments and treatments in 2019 and 2020. WW: well-watered; DS: drought stress; TY: Taiyuan; UR: Urumqi; YL: Yulin; ZY: Zhangye; YC: Yinchuan; BLUP: best linear unbiased prediction; Probability level: ** means significantly different for *P* <= 0.01. **A** Phenotypic description of yield traits under multiple environments and diffe rent water treatments in 2019; **B** Phenotypic description of yield traits under multiple environments and different water treatments in 2020; **C** Phenotypic performance of yield traits under multiple environments and different water treatments in 2019; **D** Phenotypic performance of yield traits under multiple environments and different water treatments in 2020. **E** Comparison of BLUP values of yield traits in 2019 and 2020 under different treatments

After evaluating the grain yield (GY) under well watered (WW) and drought stress (DS) in multiple environments over two years, GY was shown to follow a normal distribution (Fig. 1A-B). In 2019, the coefficient of variation(CV) of GY was significantly higher under DS than under WW conditions in the same environment (*p* < 0.01) (Fig. 1C). A similar trend was observed for GY in 2020 (Fig. 1D). In addition, analysis of variance based on GY at five locations over two years under the two water-management conditions showed that genotype effect, environment effect, and the interaction between genotype and environment were significantly different (*p* < 0.01); genotype, environment, and their interaction separately accounted for 34.96 34.48, and 24.84% of the total variation in AM115 and 41.34, 34.46, 33.07% of the total variation in AM180, respectively (Table S2). These results suggested that the difference in GY was mainly influenced by genotype, followed by environmental effects and the interaction between genotype and environment. The BLUP values of GY in 2019 and 2020 showed a similar trend; that is, GY under DS was significantly lower than that under WW (*p* < 0.01). GY declined to 29.96% in 2019 and to 24.17% in 2020, suggesting a moderate level of stress (Fig. 1E). In both populations, the coefficients of variation of GY at the five locations under DS were greater than those under normal irrigation in both years (Table 1). H^2^ of GY ranged from 71% in 2019DS to 82% in 2019WW, where the H^2^ was higher in WW than in DS (Table 1). This indicated that the drought treatment increased the difference in GY and decreased the genetic effect by inducing a stronger environmental effect.

**Table 1:** Descriptive statistical analysis of grain yield and estimated heritability on a line-mean

**Treatment**	**Location**	**Range**	**Mean ± SD**	**CV(%)**	**H**^**2**^**(%)**
2019WW	BLUP	3684.45-8687.4	6374.85±1002.9	15.73	82
	TY	1672.05-7762.95	4521.15±1282.5	28.37	
	UR	1245-10371.75	6036.45±1938.15	32.11	
	YL	2629.95-13241.85	7166.4±1680.45	23.45	
	ZY	3538.2-11444.7	6939±615.3	22.05	
	YC	2228.55-10993.65	7210.95±1632.6	22.64	
2019DS	BLUP	2771.25-6165.75	4464.75±678.3	15.19	74
	TY	868.8-7321.05	3282.45±1293	39.39	
	UR	345.3-7497.3	2958.3±1422.45	48.08	
	YL	1899.9-11162.85	5756.4±1574.55	27.35	
	ZY	846.6-8694.75	4439.1±1550.7	34.93	
	YC	957.75-9595.35	5887.35±1507.35	25.6	
2019DRI		0.42-1.03	0.70±0.12	18.26	69
2020WW	BLUP	2810.4-8615.7	6210±1240.65	19.9	84
	TY	417.75-9338.55	5590.35±1708.95	30.57	
	UR	1601.85-9286.2	6283.65±1778.7	28.31	
	YL	1708.35-9726.3	6283.65±1601.7	25.54	
	ZY	2166.45-9255.45	6126.45±1579.35	25.78	
	YC	2140.2-10313.25	6779.7±1563.6	23.06	
2020DS	BLUP	2000.85-6931.8	4708.95±948.75	20.9	76
	TY	545.1-8561.7	4693.8±1728.75	36.83	
	UR	797.7-7726.65	4078.65±1484.1	36.39	
	YL	1008.15-7668.15	4424.4±1195.65	27.02	
	ZY	1062.75-8838.45	4637.4±1569.9	33.85	
	YC	2140.2-9104.1	5709.9±1414.65	24.78	
2020DRI		0.3-1.18	0.76±0.17	22.79	71

*WW* Well-watered, *DS* Drought stress, *DRI* Drought resistance index, *BLUP* Best linear unbiased prediction, *TY* Taiyuan, *UR* Urumqi, *YL* Yulin, *ZY* ZhangYe, *YC* Yinchuan, *Mean* Arithmetic mean, *Range* The range of data, *SD* Standard deviation, *CV* Coefficient of variation, *H*^*2*^ Broad-sense heritability.

### Analysis of genetic background for the AM201 panel

**Fig. 2:**
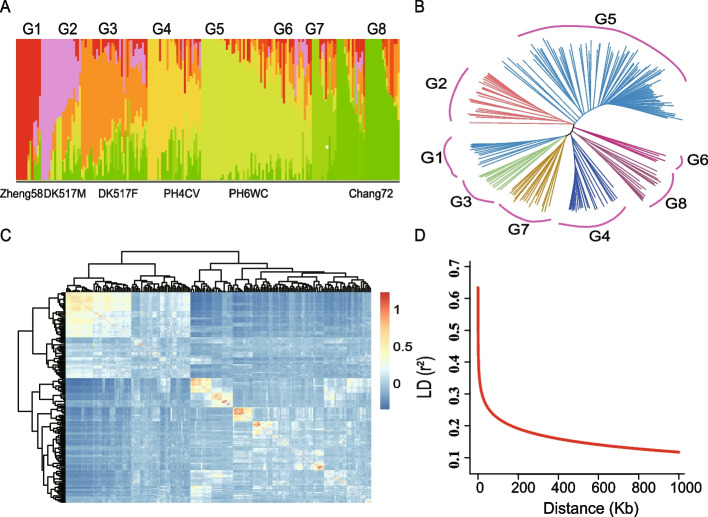
Population characterization of AM201. **A** Population structure of AM201; **B** The phylogenetic tree of AM201 was constructed with 42,003 SNPs; **C** Cluster tree and heat map showing the genetic distance of all inbred lines; **D** Linkage disequilibrium (LD) decay of AM201 (R^2^ > 0.2)

After detecting the genotype of the 201 germplasm (AM201) using the maize 6H60K SNP chip, 42,003 SNPs distributed on ten chromosomes were reserved by deleting those SNPs with missing values > 0.25 and MAF < 0.05. Among these, the number of SNPs was highest on chromosome 1 and lowest on chromosome 10, with 6,761 and 2,799 SNPs, respectively. Marker density ranged from 44.53 to 53.31 kb, with an average density of 48.36 kb (Fig. S1). Population structures were constructed using PLINK and the ADMIXTURE software, and the inbred lines were divided into eight groups according to the error rate of cross-verification and the optimal K value (Fig. 2A). The phylogenetic tree also showed eight distinct groups, in which six classical elite inbred lines (Zheng58, DK517M, DK517F, PH4CV, PH6WC, and Chang7-2) were distributed into six different subgroups (Fig. 2A-B), indicating that the groups were suitably divided. The heat map of the kinship matrix showed that most germplasms had no direct or close relationships, and several test materials were closely related to each other. The resulting matrix of kinship relationships was used to correct some of the related materials in the association analysis (Fig. 2C). In addition, the LD decay distance was 150 kb when *R*^2^ = 0.2 (Fig. 2D) and was used for candidate gene prediction during GWAS.

### GWAS for DRI and GY in maize

**Fig. 3:**
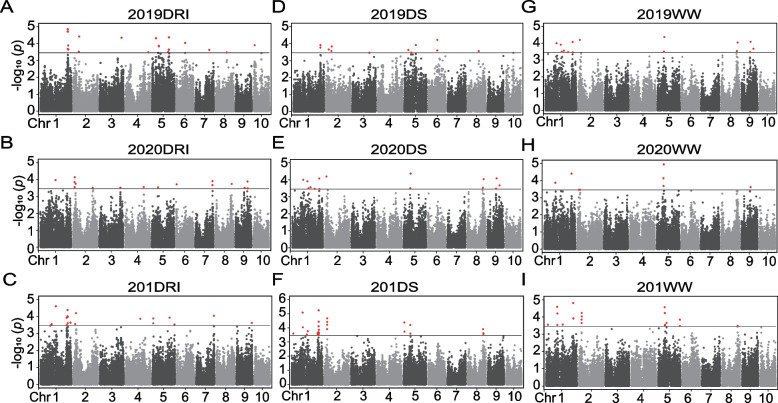
Genome-wide association results for yield and drought resistance index under different treatments in AM115, AM180, and AM201 populations. WW: well-watered; DS: drought stress; DRI: drought resistance index. **A** 2019DRI; **B** 2020DRI; **C** 201DRI; **D** 2019DS; **E** 2020DS; **F** 201DS; **G** 2019WW; **H** 2020WW; and (**I**) 201WW

To minimize the effect of environmental variation, BLUP values for GY across the five environments and drought resistance index (DRIs) were calculated for association studies using FarmCPU with a mixed linear model with kinship (K matrix) and population structure (Q matrix) to avoid spurious associations. A total of 147 associated SNPs were identified: 19, 16, 19, 12, 15, 28, 10, 8, and 20 SNPs in 2019DRI, 2020DRI, 201DRI, 2019DS, 2020DS, 201DS, 2019WW, 2020WW, and 201WW, respectively (Fig. 3). The phenotypic explained variation (PVE) at each associated site (R^2^) ranged from 0.01% to 22.19%, with an average of 3.84%. Among the 54 associated SNPs with DRIs, the PVE ranged from 0.04% to 22.19%, with an average of 4.03% and two of them were co-localized in traits 2020DRI and 201DRI, including Affx-291431276 and Affx-159033091, on chromosomes 1 and 2, respectively. In addition, six associated loci, Affx-291444080, Affx-88979175, Affx-291376326, Affx-291391165, Affx-291431276, and Affx-291405703, were co-localized in traits 2020DS and 201DS, and Affx-88980407 was co-localized in traits 2019DS and 201DS. For both conditions, the SNPs Affx-291423895, Affx-291405703, Affx-93144141, Affx-159071589, and Affx-291380961 were associated with WW, DS, and DRI (Fig. S2, Table S3). Furthermore, 18 SNPS associated with DS and DRI explained more than 10% of the phenotypic variation and were considered major effect genes (Table S3).

**Fig. 4:**
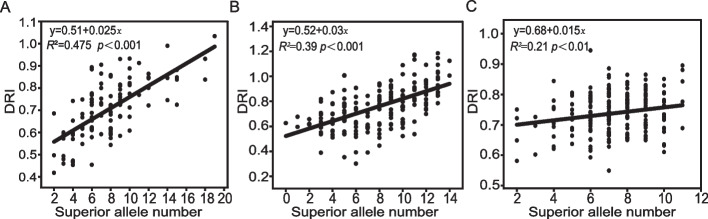
The fitting curve constructed by the number of superior alleles of significant SNPs and drought resistance index. DRI: drought resistance index. **A** 2019DRI; **B** 2020DRI; and (**C**) 201DRI

Estimation of the distribution frequency of superior alleles in different populations revealed that the average percentage of superior alleles was 39.52% (variation: 8.70–87.83%) (Table 2). Among the 54 significant loci, only 14 loci had a superior allele frequency of > 50%. After examining the cumulative effect, DRI increased with an increasing number of superior alleles for 2019DRI, 2020DRI, and 201DRI, but the largest DRI did not exceed 1.2, even if nearly 20 superior alleles were assembled (Fig. 4A-C). These results suggested that drought resistance may be improved by accumulating superior alleles; however, a large gap in drought resistance remains owing to the low frequency and effects of the superior allele in the germplasm.

**Table 2:** Significant loci information of 2019DRI,2020DRI and 201DRI

Marker	Trait	Chr	Pos	P	Superior allele	Percentage(%)
Affx-92987023	2019DRI	1	266112136	1.42E-05	A	23.48
Affx-92994854	2019DRI	1	266111259	1.96E-05	A	26.09
Affx-291418464	2019DRI	1	269708048	1.28E-04	A	29.57
Affx-291380961	2019DRI	1	266387488	2.10E-04	A	33.04
Affx-291395307	2019DRI	1	270572419	2.21E-04	A	44.35
Affx-291420667	2019DRI	2	56448250	3.80E-05	T	8.71
Affx-291377198	2019DRI	2	52443092	3.04E-04	T	26.96
Affx-291413957	2019DRI	3	214553738	4.49E-05	T	80.00
Affx-88987667	2019DRI	4	224366961	3.29E-04	A	54.78
Affx-291421562	2019DRI	5	164809159	4.26E-05	T	11.3
Affx-291423273	2019DRI	5	38235348	4.75E-05	C	87.83
Affx-291411827	2019DRI	5	65237537	1.36E-04	T	87.13
Affx-159071589	2019DRI	5	65981302	1.45E-04	T	16.52
Affx-291438415	2019DRI	5	164810587	2.28E-04	A	23.48
Affx-291433331	2019DRI	5	157593088	3.08E-04	A	20.87
Affx-291443118	2019DRI	6	85961061	9.13E-05	T	33.04
Affx-159026868	2019DRI	7	133108814	2.34E-04	T	46.96
Affx-291424956	2019DRI	8	106398090	3.33E-04	A	54.78
Affx-291424608	2019DRI	10	8358145	1.24E-04	A	73.04
Affx-291431276	2020DRI	1	148554946	1.08E-04	A	70.00
Affx-158967521	2020DRI	2	192314231	3.07E-04	G	57.78
Affx-159033091	2020DRI	2	16244527	7.35E-05	T	65.56
Affx-291395613	2020DRI	2	15255521	2.80E-04	G	22.78
Affx-291423895	2020DRI	2	13803389	1.40E-04	T	56.67
Affx-291432872	2020DRI	2	24343395	1.77E-04	T	37.78
Affx-291426183	2020DRI	3	206590683	3.05E-04	T	45.56
Affx-291424833	2020DRI	4	182813615	2.76E-04	G	30.00
Affx-291385769	2020DRI	5	61091299	2.87E-04	G	73.33
Affx-291427365	2020DRI	6	5163713	1.92E-04	C	67.22
Affx-291414708	2020DRI	7	167539198	1.25E-04	G	50.56
Affx-291427698	2020DRI	7	167385104	2.15E-04	T	48.33
Affx-291389086	2020DRI	8	158725680	1.81E-04	A	63.33
Affx-291385908	2020DRI	9	118551197	1.31E-04	A	36.67
Affx-291428790	2020DRI	9	120035179	3.29E-04	T	48.89
Affx-93144141	2020DRI	9	90399820	3.12E-04	C	25.56
Affx-158995746	201DRI	1	263494314	9.77E-05	A	19.52
Affx-291389747	201DRI	1	264234767	2.47E-04	T	31.64
Affx-291405703	201DRI	1	107434645	2.85E-04	G	19.91
Affx-291412019	201DRI	1	254683703	1.17E-04	C	23.71
Affx-291414698	201DRI	1	292212033	2.30E-04	A	43.89
Affx-291416198	201DRI	1	254681622	3.24E-04	A	37.57
Affx-291421367	201DRI	1	263705552	1.07E-04	C	35.76
Affx-291429942	201DRI	1	94088720	3.45E-04	A	15.37
Affx-291430682	201DRI	1	263494438	4.15E-05	T	16.86
Affx-291431276	201DRI	1	148554946	2.50E-05	C	61.51
Affx-159033091	201DRI	2	16244527	2.79E-04	G	18.04
Affx-291412215	201DRI	2	24331962	6.36E-05	T	14.89
Affx-291415662	201DRI	4	142539730	1.36E-04	A	16.55
Affx-123592407	201DRI	5	6932360	1.31E-04	T	42.46
Affx-291419183	201DRI	5	166555845	1.17E-04	C	31.3
Affx-291433378	201DRI	5	214757234	2.91E-04	A	43.86
Affx-291442499	201DRI	5	6679228	2.53E-04	C	42.41
Affx-291399008	201DRI	7	172865984	9.13E-05	C	10.02
Affx-291422152	201DRI	9	149935076	2.38E-04	G	26.94

Percentage (%): estimated by the ratio of the number of superior alleles for each stable locus within different groups with the number of all inbred lines; *Pos* Position; *P P*-value

### Identification of candidate genes by integrating the transcriptome

**Fig. 5:**
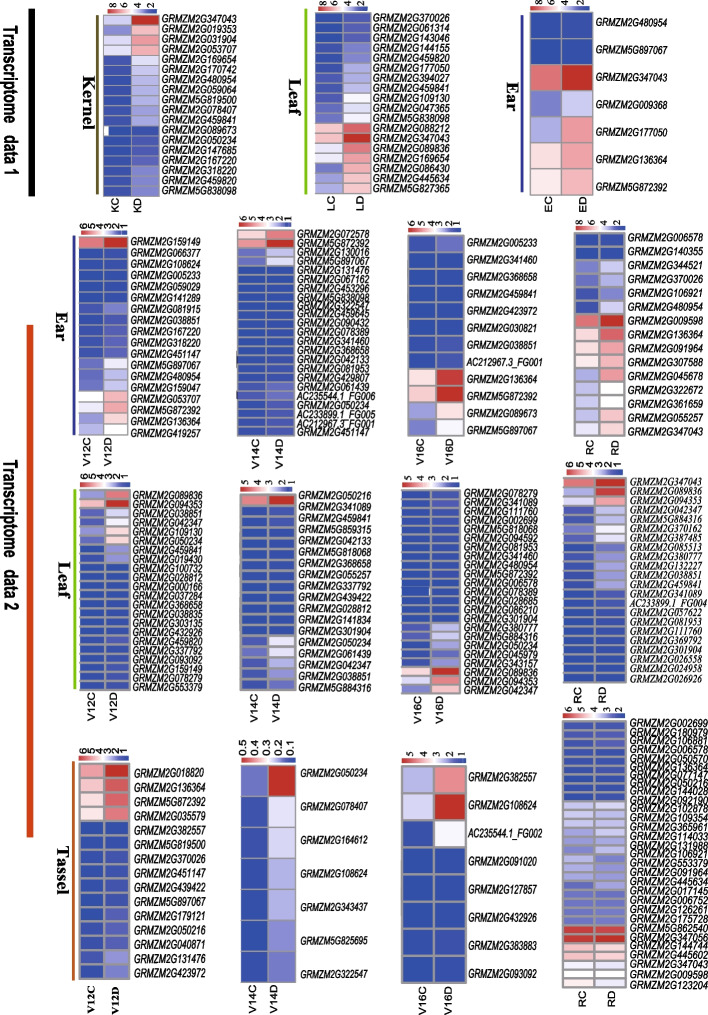
Dynamic expression patterns of DRI and DS candidate genes co-expressed with transcriptome data. C: control treatment; D: drought treatment; V12: Twelfth leaf; V14: Fourteenth leaf; V16: Sixteenth leaf

To recommend candidate genes for drought resistance, candidate genes in the confidence region were conferred according to the 150 kb LD decay distance. Herein, the genes located 150 kb around the significant sites were defined as candidate genes. Thus, 2092 candidate genes were obtained for GY under WW, GY under DS, and DRI. By integrating two public transcriptome datasets (GEO accession numbers: GSE132113 and GSE71723), including three tissues (leaf, ear, and kernel) at four developmental stages (V12, V14, V18, and R1) and three tissues (leaf, ear, and tassel branch) at two stages (V9 and 5DAP) under DS from NCBI (https://ncbi.nlm.nih.gov/), 41 and 265 differentially expressed genes were acquired as candidates, respectively (Table S4 & S5, Fig. 5); of these, 22 differentially expressed genes were common and considered core candidate genes (Fig. S3, Table 3).

**Table 3:** Candidate genes and their annotations of DRI and DS

SNPID	GeneID	Trait	Chr	Module	Start	End	PVE(%)	*P*-value	Pathway	Annotation
Affx-291427547	*GRMZM2G053707*	2020DS	5	M1	67656007	67656832	16.98	4.26E-05		--
Affx-291389747	*GRMZM2G009368*	201DS,201DRI	1	M2	264332024	264335078	5.06	2.47E-04	Ethylene signaling pathway	NRAM Pmeta lion transporter family protein
Affx-291395613	*GRMZM2G050234*	2020DRI	2	--	15139189	15141339	11.32	2.80E-04		DMR6-LIKE OXYGENASE 1
Affx-291441780	*GRMZM2G078407*	2020DS	1	--	221014499	221015760	9.52	3.35E-04		
Affx-159033091	*GRMZM2G086430*	201DRI,2020DRI	2	M3	16359701	16365072	22.18	7.35E-05	Phosphorus deficiency stress	spx8 - SPX domain-containing membrane protein8
Affx-291442499	*GRMZM2G089673*	2020DRI,201DS	5	M4	6575359	6577757	0.24	2.53E-04		--
Affx-291389747	*GRMZM2G109130*	201DS,201DRI	1	M5	264266381	264271190	5.06	2.47E-04	Resistancetopests	lipoxygenase
Affx-291420667	*GRMZM2G136364*	2019DRI	2	M6	56316542	56317722	2.84	3.80E-05	Drought stress response	Lipid binding protein
Affx-291389086	*GRMZM2G167220*	2020DRI,2020DS	8	M7	158737677	158740474	12.39	9.04E-05	Drought stress response	Cytokinin oxidase 3
Affx-291426183	*GRMZM2G169654*	2020DRI	3	M8	206678255	206680041	0.16	3.05E-04	ABA signaling pathway	ereb126 - AP2-EREBP-transcription factor 126
Affx-291399008	*GRMZM2G177050*	201DRI	7	M9	172920911	172923332	0.26	9.13E-05	Drought stress response	cipk29 - calcineurin B-like-interacting protein kinase29
Affx-158967521	*GRMZM2G318220*	2020DRI	2	--	192420968	192422652	15.85	3.07E-04	--	--
Affx-291414698	*GRMZM2G347043*	201DRI	1	M10	292162441	292164227	0.15	2.30E-04	Transcriptional regulation	nactf49 - NAC-transcription factor 49
Affx-291430631	*GRMZM5G838098*	201DS	1	M11	16782463	16783895	0.81	2.79E-04	JA and SA signaling pathway	zim27 - ZIM-transcription factor 27
	*GRMZM2G445634*	201DS	1	M12	16791540	16792462	0.19	2.51E-04	JA and SA signaling pathway	zim16 - ZIM-transcription factor 16
Affx-291427698	*GRMZM2G459841*	2020DRI	7	M13	167382574	167383555	21.53	2.15E-04	--	--
Affx-291435219	*GRMZM2G480954*	201DS	1	M14	263817609	263818594	3.77	3.25E-04	Drought stress response	oleosin4
Affx-291431276	*GRMZM5G819500*	201DS,2020DS	1	--	148373492	148374331	0.26	2.50E-05	--	--
Affx-291413957	*GRMZM5G827365*	2019DRI	3	M15	214559544	214561574	0.80	4.49E-05	--	F-box domain containing protein
Affx-291377201	*GRMZM2G370026*	2020DS	1	M16	179860588	179861190	16.19	2.75E-04	--	bzip31 - bZIP-transcription factor 31
Affx-291421562	*GRMZM5G872392*	2019DRI	5	M17	164895210	164898398	1.32	4.26E-05	Drought stress response	sweet15b - sugars will eventually be exported transporter15b
Affx-159071589	*GRMZM5G897067*	2019DS	5	M18	81896162	81897912	0.49	1.45E-04		--

*WW* Well-watered, *DS* Drought stress, *DRI* Drought resistance index, *PVE(%)* Phenotypic variation explanation

**Fig. 6:**
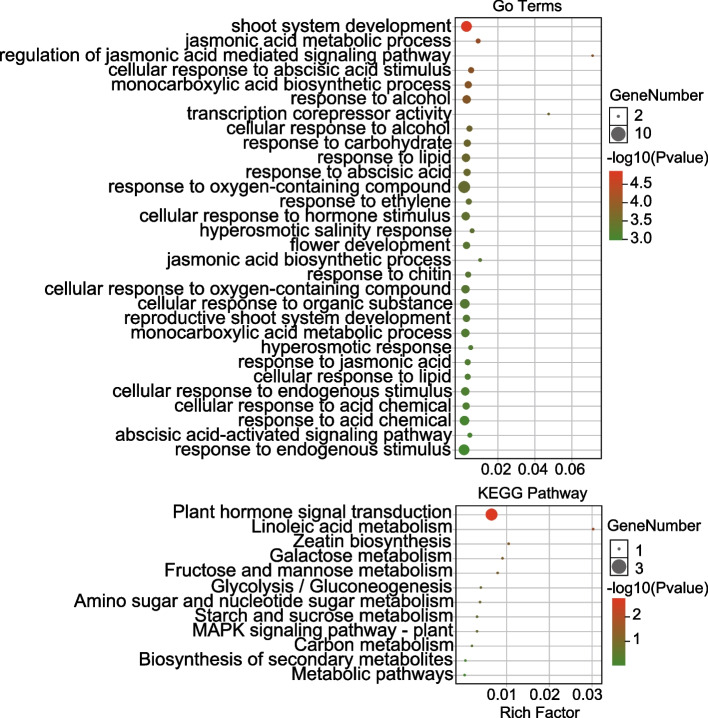
KEGG and GO functional enrichment analyses of 22 candidate genes. The size of the circle represents the number of enriched genes, and the *P*-value indicates enrichment significance. *P* < 0.01

Further annotation of these 22 candidate genes using GO and KEGG analyses revealed that they were enriched in plant hormone signal transduction and participated in several biological processes, including response to alcohol, shoot system development, reaction to oxygen-containing compounds, jasmonic acid (JA)- and salicylic acid (SA)-mediated signaling pathway regulation, response to carbohydrates, response to stress, and response to water shortage. In addition, molecular functions were enriched in transcription corepressor activity, metal ion binding, cation binding, and ion binding (Fig. 6, Table S6). These results suggested that hormone and transcriptional regulation play important roles to response DS.

### Protein-protein interaction networks predicted by candidate genes

Based on the STRING (v11.0) database, an interaction network was predicted for the 22 core candidate genes. Of these, 18 were used to construct protein-protein networks that interacted with various functional proteins and with each other via an intermediate protein. The coding proteins participated in protein-protein network interactions. The protein interaction network, using the candidate genes as the core including *GRMZM2G109130*, *GRMZM2G167220*, *GRMZM2G169654*, *GRMZM2G318220*, *GRMZM2G347043*, *GRMZM5G838098*, *GRMZM2G445634*, *GRMZM2G480954*, and *GRMZM2G370026*, showed that these coding proteins belong to the lysyl oxidase, cytokinin oxidase (*CKO*), related to ABI3/VP1 (*RAV*), glutathione peroxidase, N-acetylcysteine (*NAC)*, *JAJZ1*, *OLE*, and basic leucine zipper (*bZIP*) transcription factor families, respectively. These transcription factor families mainly respond to DS by regulating organic acid metabolism, oxygen-containing compound reactions, and JA and SA mediated signaling pathways.

**Fig. 7:**
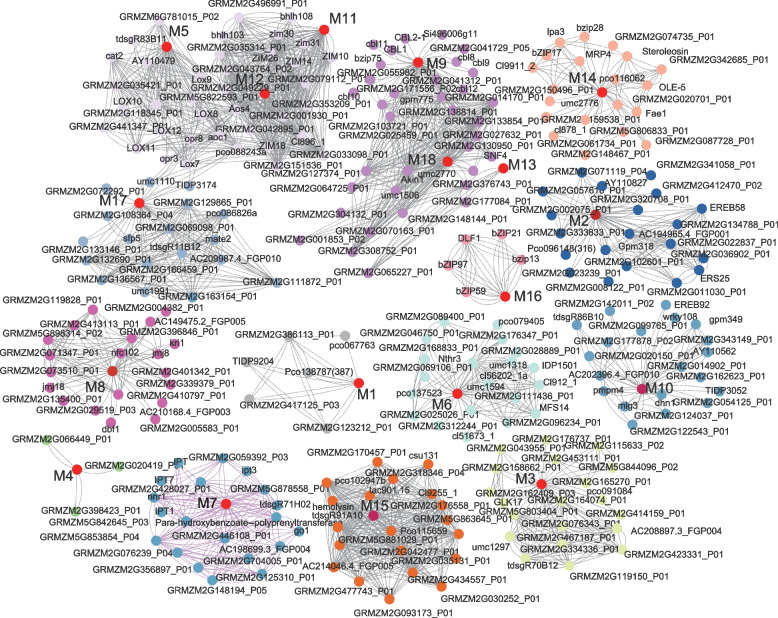
Protein–protein interaction networks of candidate genes. Nodes indicate proteins and lines indicate the interaction between proteins. The red circle indicates proteins encoded by the identified candidate genes and the interactive proteins in different networks are distinguished using different colors. M1–M18 are the candidate genes encoded proteins as Table 3

Some protein-encoding candidate genes participated in responses to DS through common interacted genes between different interaction networks. For example, protein interaction networks with the core coding proteins of candidate genes *GRMZM2G109130* (M5), *GRMZM5G838098* (M11), *GRMZM2G445634* (M12), and *GRMZM5G872392* (M17) (Fig. 7) were mainly associated with biological processes involved in a network of signaling pathways, including the phytohormones JA/ethylene and SA, response to endogenous stimulus, response to hormone, and cellular response to organic substance. The interaction network of the coding proteins of candidate genes *GRMZM2G177050* (M9), *GRMZM2G459841* (M13), and *GRMZM5G897067* (M18) was mainly involved in the response to hyperosmosis, cell response to hormone stimulation, monocarboxylic acid biosynthetic process, and root and flower development, which play an important role in plant responses to biotic and abiotic stresses. These results provide a deeper understanding of the interactions between different drought response factors.

## Discussion

### Co-localization of associated loci with those from previous studies

Drought is an abiotic stress with great influence on the growth and development of maize [44]. In particular, subjection of maize to DS at the flowering stage leads to significant yield reduction [45, 46]. Therefore, understanding the phenotypic variation under drought and the genetic basis response to drought in maize is crucial for determining its drought sensitivity. Many studies have applied GWAS to elucidate the genetic mechanism of drought-resistant traits, and some results have been achieved [21, 47, 48]. In most published studies, the regulatory mechanism of drought resistance has been analyzed through physiological indicators such as chlorophyll content during the seedling stage [23, 24, 48, 49]. However, the Drought Resistance Index (DRI) calculated using yield (GY) under normal and stress conditions, which is an effective indicator for evaluating drought resistance of germplasm during breeding process [50–52], but is rarely used to understand the regulatory mechanism of drought resistance.

In this study, a GWAS was conducted for GY and DRI using an associated population consisting of 201 inbred maize lines that formed three sets of association panels according to actual plant information. In 2019 and 2020, 55 SNP markers were significantly associated with GY under DS conditions, 54 for DRI, and 38 for GY under WW conditions. Most of the associated SNPs indentified under WW and DS is different, which also happened for the comparison of the GWAS results under stressed and normal treatments [19]. These results indicate that the identified QTL may vary under different environmental conditions within the same population [44, 53]. In extreme cases, consensus loci were not detected for the same traits under different treatments, which is similar to the results of previous studies [54]. Here, 12 associated SNPs were shared between GY or DRIs from different years or treatments.

In addition, some associated SNPs were consistent with QTLs detected by multi-population linkage mapping or association analysis. On chromosome 1, a significant SNP (Affx-291414698) associated with 201DRI was located 45 kb from the nuclear NAC-transcription factor 49 (*NAC49*), which have been proved that *ZmMPK5* phosphorylates the subdomain Thr-26 in *NAC49* and enhances SOD activity to improve the oxidative stress tolerance of maize [55]. Affx-88980407 associated with 2019DS, Affx-291432872 for 2019DRI, Affx-291412215 for 201DRI, and other four SNPs (Affx-88980402, Affx-88980404, Affx-88980407 and Affx-291389339) for 201DS, were co-located in the MQTL10 interval with a size of 22.38-28.27Mb on chromosome 2 [53]. Affx-88984415 associated with 2019WW located on chromosome 3, was co-located in the QTL named qWW-GY7-1 interval with a size of 17.7-19.8Mb located using a doubled happy (DH) population consensus of 217 lines [56] . Affx-291377201 associated with 2020DS located on chromosome 1, was located in the region of mQTL_GY_1a with a interval of 161.07-183.29Mb by using three tropical maize populations [57], also shared the same QTL region on 1.06 for GY across WW and WS environments by using RFLP markers in a F3 population of tropical maize [58] and identified a cluster of QTL on bin 1.06 related to GY and other yield contributing traits under drought as well as WW conditions in Mexican and African environments [59]. The above significant SNP can provide a reference for further research and exploration of maize drought resistance molecular breeding.

### Candidate genes for drought resistance

By combining GWAS and two sets of transcriptome data, a total of 41 (Table S4 & Table S7) and 265 (Table S5 & Table S8) genes were mined and annotated, respectively. These genes participated in the regulation of the JA-mediated signaling pathway, response to oxygen-containing compounds, response to endogenous stimuli, response to ABA and JA metabolic process, and response to stress. In addition, some transcription factors, such as nuclear factor-YA, C2C2-DNA-binding one zinc finger, ethylene responsive factor (*ERF*), *B3, bZIP, E2f-dp, OVATE* family protein, *NAC*, myeloblastosis, and basic helix–loop–helix were associated with stress. Candidate genes, including *GRMZM2G322672* (*EREB37*), *GRMZM2G026926* (*ERF*), and *GRMZM2G169654* (*RAV*), belong to the *AP2/EREBP* family, which has been cloned in crops, and performed important roles to response drought have been elucidated [53, 58]. In addition, the candidate genes *GRMZM2G370026* (*bZIP31*), *GRMZM2G140355* (*bZIP80*), and *GRMZM2G006578* (*bZIP7*) belong to the *bZIP* family, which participates in various secondary metabolite synthesis pathways and protects plants from damage and stress by accumulating various metabolic substances [60–64].

Seven of these associated SNPs explained more than 10% of the phenotypic variation, and the related putative genes that showed significantly differential expression under DS conditions were *GRMZM2G086430* (Affx-159033091), *GRMZM2G167220* (Affx-291389086), and *GRMZM2G050234* (Affx-291395613) (Table 3). GRMZM2G086430 (SPX8) is a membrane protein containing the domain SPX8, where the promoter region of *ZmSPX*s is rich in biological/abiotic stress elements [65]. GRMZM2G167220 is a cytokinin dehydrogenase involved in the synthesis of tolerance-related metabolites during deep sowing of maize, which is an important method to improve drought resistance [66]. *GRMZM2G050234* is translated into an oxidoreductase that is involved in the REDOX process [67, 68]. Another candidate gene, *GRMZM2G347043* (*ZmNAC49*), with a low PVE for 201DRI, has demonstrated that drought tolerance in maize can be improved by reducing stomatal density during the seedling stage, enhancing oxidative stress tolerance and responding to the mitogen-activated protein kinase signaling pathway [55, 57, 69–71]. *ZmNAC49* overexpression enhances drought tolerance of maize seedlings [72]. Thus, the above genes may be used for further research to understand drought resistance in maize.

### A large gap for improving drought resistance in maize

Enhancing drought tolerance in plants is a multifaceted process. While genetic engineering is one approach, selecting and accumulating favorable alleles of crucial stress-tolerance genes can also be a promising strategy for crop improvement [22]. In this study, the assembly of superior alleles illustrated that the number of excellent alleles for DRI was positively correlated with the DRI value. Among the 54 significant loci, only 14 loci contained superior alleles in more than 50% of materials in AM201, and the largest DRI value still did not reach 1.2 (Fig. 4A-C). This indicates that enriching superior alleles in the existing drought-resistant germplasm is not sufficient, which is consistent with a previous study [46]. Sensitivity to drought increases concomitantly with increasing maize yield [73]. The synergy between drought resistance and yield may be one reason why extremely drought-resistant materials are not frequently used in breeding. In other words, some drought resistance-related superior alleles will be fixed and others will be lost during the breeding process. Therefore, it is of great significance to study the synergistic effect between drought resistance and yield and some progress has been made in plants.

For example, genetic engineering may be used to manipulate or delete genes, such as *ZmMPKL1* and its homologs, to maintain yield stability by reducing the drought sensitivity of maize [24]. *ZmGLK44* promotes tryptophan levels by activating the expression of tryptophan biosynthetic genes, which further enhances ABA signaling and water use efficiency to help plants resist drought [47]. The mechanisms driven by transposable element-inverted repeat structures pave the way for genome manipulation to design crops with high stress tolerance and high yields for the future [74]. These results indicated that drought resistance might be improved by filling the gap between drought resistance and yield with synergistic effects.

## Conclusion

In this study, GY was investigated in associated panels under five environmental conditions with different moisture treatments for two years. A total of 147 significant SNP loci were identified using GWAS, of which 109 were associated with GY under DS and the DRI. There was a positive relationship between the number of superior alleles and DRI. Finally, 22 genes involved in cytokinin dehydrogenase activity, transcription factor activity, and ion binding were considered candidate genes by integrating transcriptome data. This study provides important reference information for exploring drought-resistance mechanisms and the application of molecular marker-assisted selection for drought-resistant breeding of maize.

### Supplementary Information

**Additional file 1:** **Figure S1.** The distribution density of 42,003 SNPs on different chromosomes.

**Additional file 2:** **Figure S2.** Significant SNPs co-located in different traits. 2019DS, 2020DS, 201DS represented the results grain yield under drought stress in AM115,AM180 and AM201 respectively. And 2019WW, 2020WW and 201WS represented the results of grain yield under well water in AM115, AM180 and AM201 respectively.

**Additional file 3:** **Figure S3.** Transcriptome data 1 and 2 co-locate candidate genes.

**Additional file 4:** **Table S1.** List of 201 inbred lines.

**Additional file 5:** **Table S2.** Joint analysis of variance of yield under different treatment conditions. SS: sum of squares; SS(%): Percent of SS; MS: mean squares; *, and ** means significantly different for *P* <= 0.05, *P* <= 0.01.

**Additional file 6:** **Table S3.** Phenotypic interpretation rate of significant SNP loci in different traits. WW: well-watered, DS: drought stress; DRI: drought resistance index; Percentage (%) : estimated by the ratio of the number of superior alleles for each stable loci within different group with the number of all inbred lines; The red-marked SNP is co-located with 201DRI and 2020DRI; The blue-marked SNP is co-located with 201DS and 2020DS; The purple-marked SNP is co-located with 201DS and 2019DS; The green-marked SNP is co-located with all traits.

**Additional file 7:** **Table S4.** 41 Key differentially expressed genes in different periods and tissues under drought stress.

**Additional file 8: Table S5.** 265 key differentially expressed genes in different tissues and different periods under drought stress.

**Additional file 9:** **Table S6.** GO and KEGG enrichment analysis of 22 candidate genes.

**Additional file 10:** **Table S7.** GO enrichment for 41 differentially expressed genes.

**Additional file 11:** **Table S8.** GO enrichment for 265 differentially expressed genes.

## Data Availability

All reasonable requests for data and research materials should be addressed to the corresponding author (shutuxu@nwafu.edu.cn). Transcriptome data were obtained from the National Center for Biotechnology Information (https://www.ncbi.nlm.nih.gov/geo/query/acc.cgi?acc=GSE132113 and https://www.ncbi.nlm.nih.gov/geo/query/acc.cgi?acc=GSE71723). The data have been uploaded to the National Agricultural Science Data Center (Project number: 2018YFD0100200). Genotype and phenotype data serial number A1C6936557C165AA6602839C316D1BCB and BF5CA18FD7F1AFF75CAFBEDEFE3D4525, respectively.

## Abbreviations

DS: Drought stress
WW: Well-watered
CK: Control check
TY: Taiyuan
UR: Urumqi
YL: Yulin
ZY: ZhangYe
YC: Yinchuan
DAP: Days after pollination
G: Genotype
E: Environment
G $\times$ E: Interaction between genotype and environment
V9: Ninth leaf
V12: Twelfth leaf
V14: Fourteenth leaf
V16: Sixteenth leaf
VT: Tasseling
V: Vegetative stages
R: Reproductive stages
DRI: Drought resistance index
GY: Grain yield
GWAS: Genome-wide association study
ASI: Anthesis and silking interval
DC: Drought coefficient
LD: Linkage disequilibrium
RFLP: Restriction fragment length polymorphism
SNP: Single nucleotide polymorphism
QTL: Quantitative trait loci
ABA: Abscisic acid
PVE: Phenotypic variation explanation
JA: Jasmonic acid
SA: Salicylic acid
SOD: Superoxide dismutase
REDOX: Oxidation-reduction reaction
NAC: N-acetylcysteine
ERF: Ethylene-responsive factor
RAV: Related to ABI3/VP1
BLUP: Best linear unbiased prediction
SD: Standard deviation
CV: Coefficient of variation
H^2^: Broad-sense heritability
GEO: Gene Expression Omnibus
GO: Gene Ontology
KEGG: Kyoto Encyclopedia of Genes and Genomes
PCA: Principal component analysis
NCBI: National Center for Biotechnology Information
