# Supplementary figures and images for: Molecular mechanisms of drought resistance using genome-wide association mapping in maize (Zea mays L.)
Source: BMC Plant Biol. 2023 Oct 6;23:468. doi: 10.1186/s12870-023-04489-0 (PMC10557160; doi:10.1186/s12870-023-04489-0)

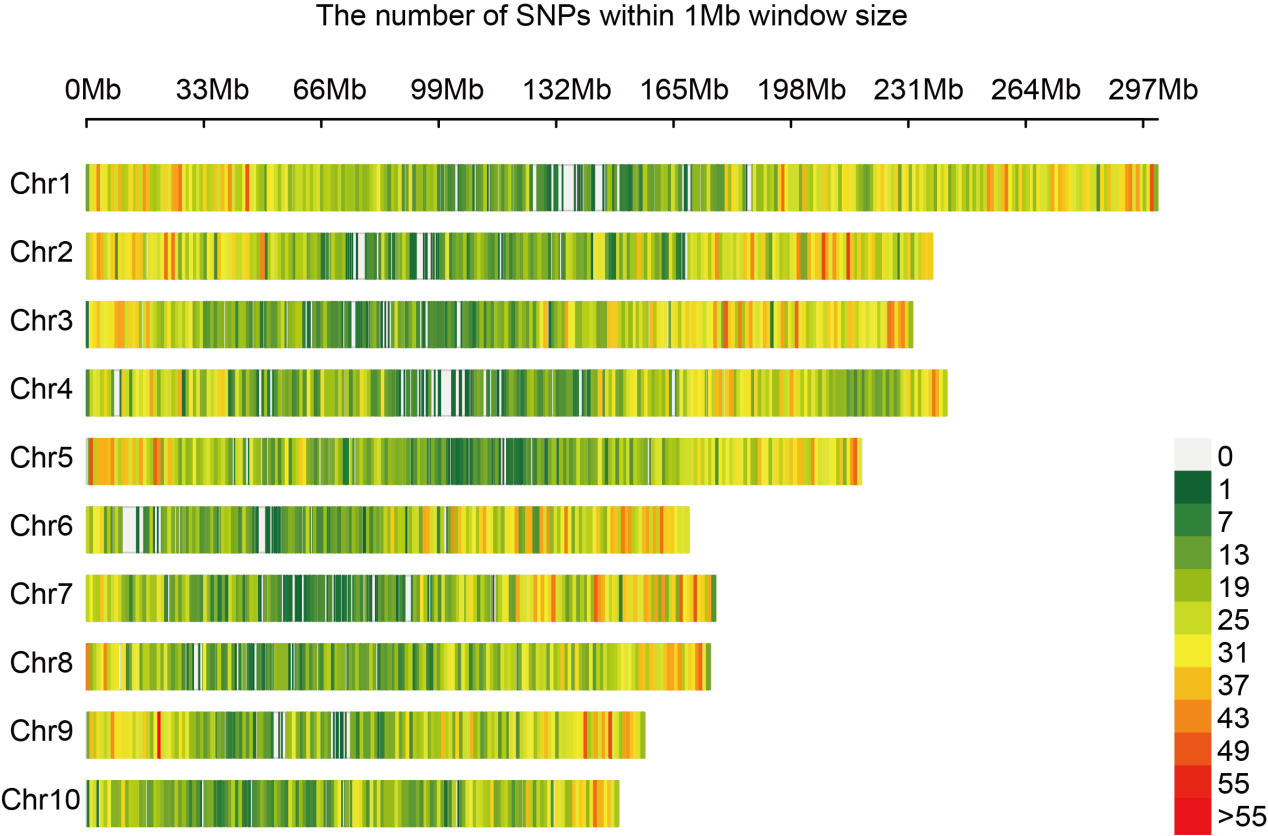


**Figure S1 The distribution density of 42,003 SNPs on different chromosomes.**

Supplement: Supplementary file 1 — Additional file 1: Figure S1. The distribution density of 42,003 SNPs on different chromosomes. [file 12870_2023_4489_MOESM1_ESM.docx]

**
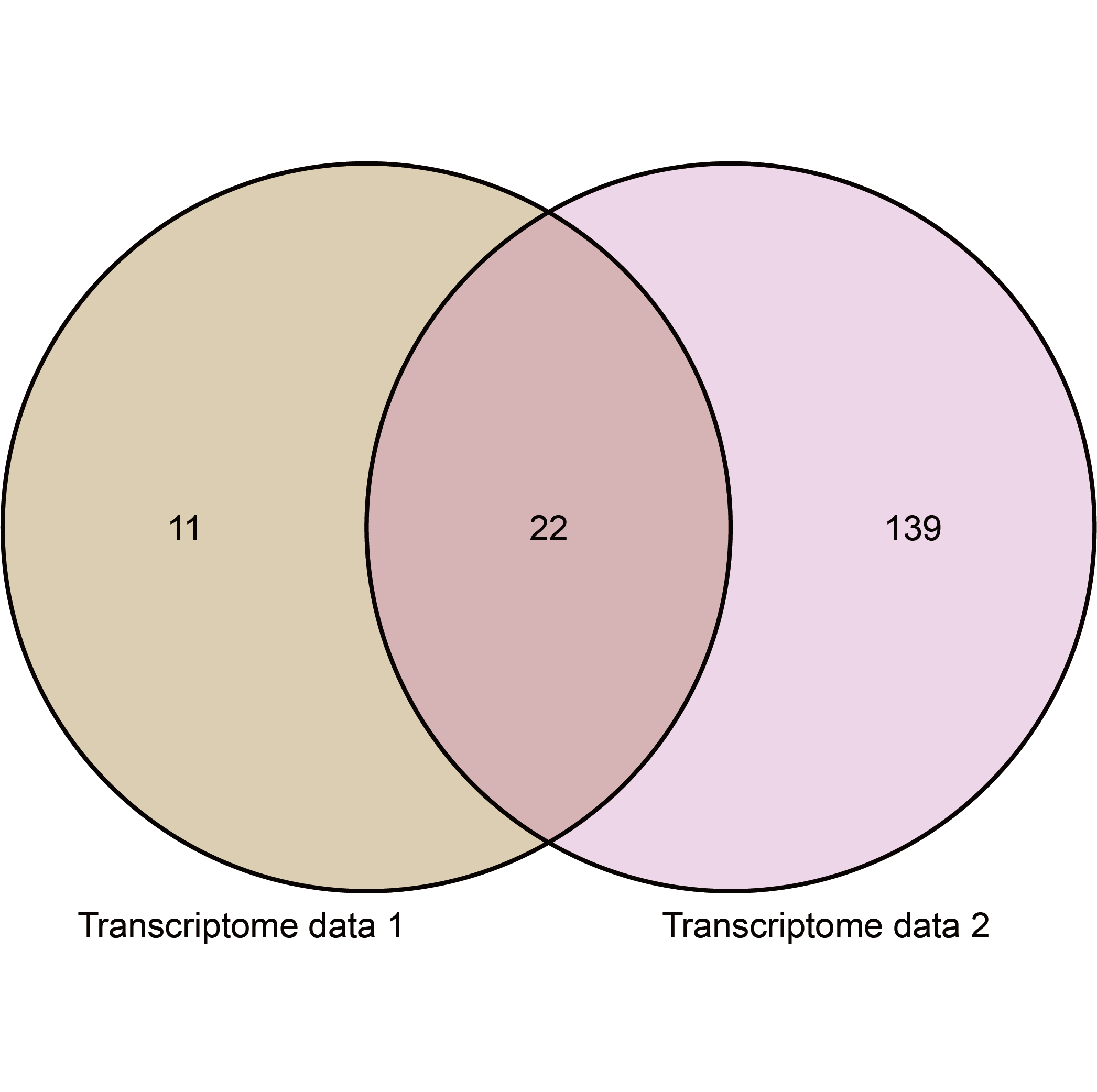
**

**Figure S3 Transcriptome data 1 and 2 co-locate candidate genes.**

Supplement: Supplementary file 3 — Additional file 3: Figure S3. Transcriptome data 1 and 2 co-locate candidate genes. [file 12870_2023_4489_MOESM3_ESM.docx]
