# Supplementary material for: Molecular mechanisms of drought resistance using genome-wide association mapping in maize (Zea mays L.)
Source: BMC Plant Biol. 2023 Oct 6;23:468. doi: 10.1186/s12870-023-04489-0 (PMC10557160; doi:10.1186/s12870-023-04489-0)
Supplement: Supplementary file 2 — Additional file 2: Figure S2. Significant SNPs co-located in different traits. 2019DS, 2020DS, 201DS represented the results grain yield under drought stress in AM115,AM180 and AM201 respectively. And 2019WW, 2020WW and 201WS represented the results of grain yield under well water in AM115, AM180 and AM201 respectively. [file 12870_2023_4489_MOESM2_ESM.docx]

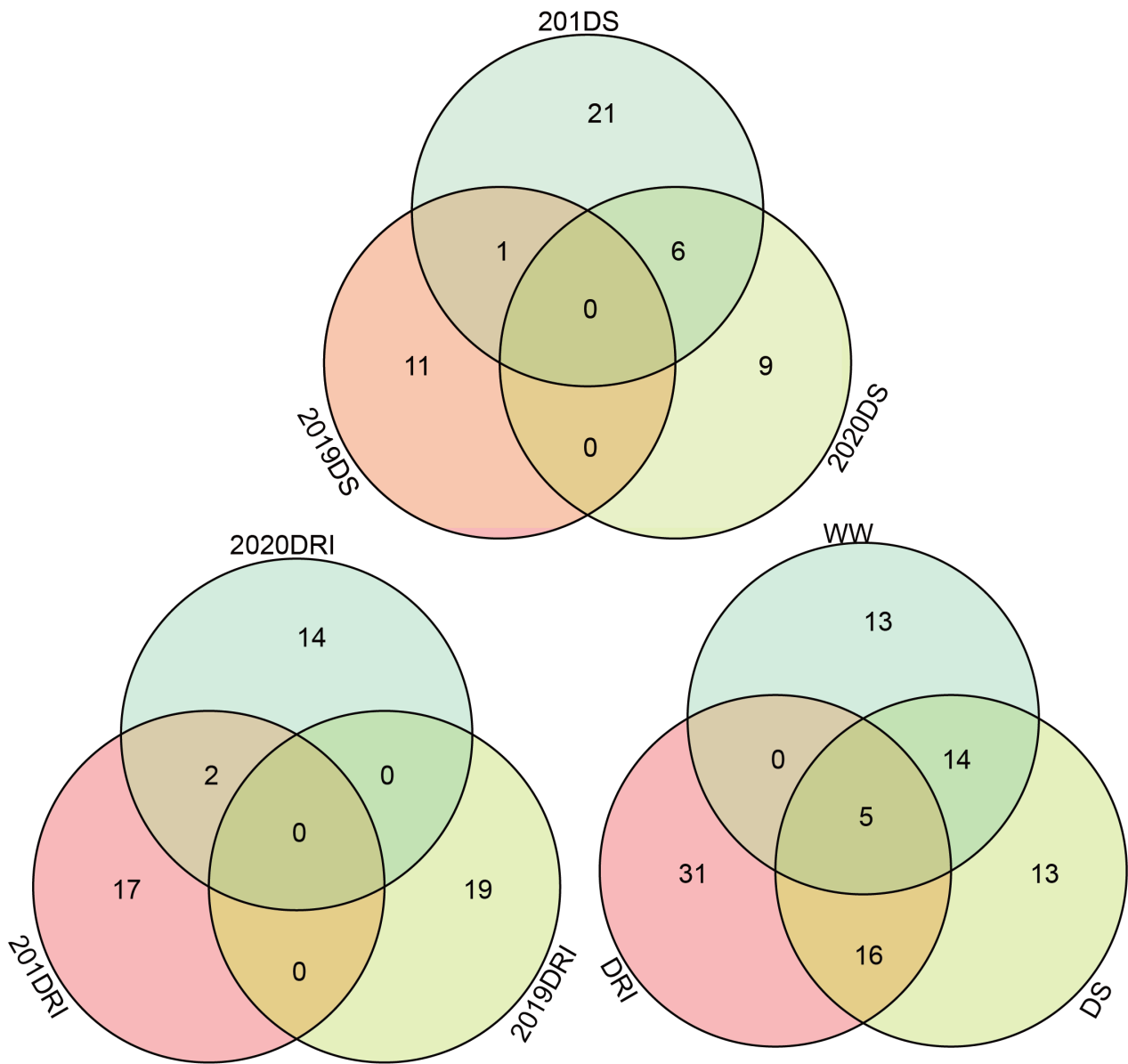


**Figure S2 Significant SNPs co-located in different traits.** 2019DS, 2020DS, 201DS represented the results grain yield under drought stress in AM115,AM180 and AM201 respectively. And 2019WW, 2020WW and 201WS represented the results of grain yield under well water in AM115, AM180 and AM201 respectively.
